# Safety of Non-Steroidal Anti-Inflammatory Drugs in the Elderly: An Analysis of Published Literature and Reports Sent to the Portuguese Pharmacovigilance System

**DOI:** 10.3390/ijerph19063541

**Published:** 2022-03-16

**Authors:** Cristina Monteiro, Samuel Silvestre, Ana Paula Duarte, Gilberto Alves

**Affiliations:** 1 UFBI—Pharmacovigilance Unit of Beira Interior, University of Beira Interior, 6200-506 Covilhã, Portugal; apcd@ubi.pt (A.P.D.); gilberto@fcsaude.ubi.pt (G.A.); 2 CICS-UBI—Health Sciences Research Centre, University of Beira Interior, 6200-506 Covilhã, Portugal; samuel@fcsaude.ubi.pt; 3 ESALD-IPCB—Dr. Lopes Dias School of Health, Polytechnic Institute of Castelo Branco, 6000-767 Castelo Branco, Portugal

**Keywords:** adverse drug reactions, non-steroidal anti-inflammatory drugs, safety, elderly

## Abstract

Non-steroidal anti-inflammatory drugs (NSAIDs) are the most frequently used agents to treat musculoskeletal disorders (principally by the elderly), thus raising the risk of adverse drug reactions (ADRs). This work aims to monitor NSAIDs safety profile in older people by using literature and pharmacovigilance data. Published clinical studies reporting the NSAIDs safety in elderly patients (age ≥ 65) were identified by a literature search and were then deeply analyzed. In addition, suspected ADRs reports submitted to the Portuguese Pharmacovigilance System (PPS) involving patients aged ≥65 with at least one NSAID as suspected drug were explored in detail. Most studies concluded that the risk of gastrointestinal, cardiovascular, and renal ADRs was significantly lower with cyclooxygenase-2 (COX-2)-selective NSAIDs use than with nonselective NSAIDs. The PPS data analysis showed that serious gastrointestinal ADRs occurred mostly in patients taking more than one NSAID and/or another concomitant drug that increases the incidence of these events, in the absence of gastroprotection. The results suggest that while NSAID toxicity is well understood, their safe use needs to be monitored in clinical practice. Furthermore, the pharmacovigilance data analyzed also showed that monitoring NSAIDs use in elderly remains essential to mitigate the associated risks, especially in those with comorbidities and under polytherapy.

## 1. Introduction

The use of non-steroidal anti-inflammatory drugs (NSAIDs) for a wide range of rheumatic conditions and other musculoskeletal disorders is increasing. This is in part due to the growing number of elderly patients who constitute the main users of these drugs [1,2]. Despite their relevant efficacy, NSAIDs must be used with caution in older people due to the high risk of potentially serious and life-threatening adverse effects [1]. NSAIDs constitute a group of therapeutic agents with diverse structural and pharmacological profiles but a similar mechanism of action. They inhibit the cyclooxygenase-1 (COX-1) and cyclooxygenase-2 (COX-2) enzymes that are involved in biosynthesis of inflammatory mediators such as prostaglandins, which also play a protective role in multiple physiological functions involving the gastrointestinal, renal, and cardiovascular systems among others [1,3]. Therefore, it is not surprising that NSAIDs trigger some important deleterious effects, and in older people they have been implicated in 23.5% of hospital admissions due to adverse drug reactions (ADRs) [4]. Although these drugs are generally associated with mild gastrointestinal adverse events on short-term use, more serious adverse events such as gastrointestinal ulceration or bleeding may arise under long-term use [5]. In fact, gastrointestinal toxicity can occur with all NSAIDs, which may be of particular concern when treating older patients. However, gastrointestinal adverse events may be reduced by taking a concomitant gastroprotective agent [2]. In addition, older patients have usually other comorbidities, mostly cardiovascular diseases and/or decline in renal function, and for these reasons they frequently need to use other drugs that can potentially interact with NSAIDs and consequently increase the risk of adverse cardiovascular, hematologic, and renal events [2]. The main problematic drugs in this context include selective serotonin reuptake inhibitors, corticosteroids, digitalis glycosides, diuretics, beta-blockers, calcium antagonists, angiotensin converting enzyme inhibitors, clopidogrel, low-dose acetylsalicylic acid, warfarin, and other anticoagulant agents [1,6].

With the goal of reducing serious gastrointestinal adverse effects ascribed to conventional (i.e., COX-2-nonselective) NSAIDs, highly selective COX-2 inhibitors, called coxibes, were introduced into clinical practice about 20 years ago. However, they have only limited benefit in reducing these untoward effects. In fact, the risk of serious cardiovascular and renal adverse effects remains as a major concern [1,7].

Consequently, it is important to perform a continuous monitoring of the safety of these drugs in older population by means of pharmacovigilance or other post-authorization safety studies. In this context, the identification of preventable ADRs is an important starting point to improve drug safety in elderly [8]. Moreover, due to the comorbidities they often present, elderly patients do not always participate in clinical trials, and therefore treatment recommendations for this special population are usually based on the extrapolation of evidence obtained from clinical trials conducted in healthy and younger subjects [9,10]. Currently, there are already several recent reviews addressing the use of NSAIDs in elderly [11,12,13,14,15]. However, it is important to continue monitoring the NSAIDs safety in this special population, gathering real world evidence on the occurrence of serious ADRs, the impact of concomitant drugs, and the effect of gastroprotection use. For all of these reasons, we intended to integrate the evidence from the most recent clinical studies and the reports of suspected ADRs received in a pharmacovigilance database.

In this context, we carried out a comprehensive literature review of clinical trials and observational and interventional clinical studies that report data on NSAIDs safety in the elderly. In addition, we intended to characterize in elderly patients the suspected ADRs associated with NSAIDs reported to the Portuguese Pharmacovigilance System (PPS) from 2008 to 2018. The overall aim was to conclude about the safety of NSAIDs in the elderly, considering in an integrated manner the available scientific literature and the real-world evidence obtained from pharmacovigilance activities. Secondarily, we intended to compare the extent to which the drug safety documented in the literature is reflected in the safe use of these drugs by this population.

## 2. Materials and Methods

### 2.1. Comprehensive Review

A bibliographic search was performed in different databases (Pubmed, Web of Knowledge, Medline and Cochrane Collection Plus) to identify studies addressing the safety of NSAIDs in older patients (age ≥ 65). This search considered the period between 1 January 2005 to 23 January 2020 and was performed using the following terms: (adverse reaction OR adverse event OR safety OR pharmacovigilance) AND (non-steroidal anti-inflammatory) AND (elderly OR older people OR older patient OR older person OR geriatric OR older adult) AND (Humans [Mesh]) and the filters age ≥65 and articles related with clinical studies were applied. In the process to select the studies to be included in the review, the exclusion of all studies referring to NSAIDs that had already been removed from the market was considered. In fact, the objective of using only NSAIDs currently on the market and the fact that several reviews on this subject have already been published [1,2,5,6], which were the reasons for the selection of the time period referred to. Thus, studies focused on rofecoxib and lumiracoxib were excluded [16,17].

Other criteria of exclusion were: review articles, pre-clinical in vivo studies, studies that did not describe drug-safety, studies with ambiguous design or methods, studies in which the analyzed population included people under 65 years or the results were not presented by specific age (i.e., studies that involved young adults and elderly, but the results were presented in terms of average age), studies with ocular formulations, and studies where the results were not separated by specific drug.

Observational and interventional studies were considered. Only studies published in English were included.

The outcomes considered were related to the safety of NSAIDs in older people, with the description of ADRs associated, and the conclusion about which drug is safer in the older people.

### 2.2. Analysis of Adverse Drug Reactions Reports Sent to Portuguese Pharmacovigilance System

An observational and retrospective analysis of suspected ADRs reported to the PPS was performed. The PPS is coordinated by the National Authority of Medicines and Health Products, I.P. (INFARMED).

In this analysis, it was considered only the reports involving one or more NSAIDs as suspected drug(s) in patients aged 65 or over, between the period of 2008 to 2018. Duplicates and reports that did not present the necessary information for ADRs characterization were excluded, namely those that did not mention age. The reports referring to eye drops or acetylsalicylic acid as antiplatelet agent were excluded. It is also important to note that each notification concerns a single case, but for each notification more than one suspected ADR and more than one implicated drug may be associated.

Initially, 367 reports were considered, of which 49 were duplicated, 6 did not mention age, 5 referred to eye drops and 46 involved acetylsalicylic acid at a low dosage, acting as an antiplatelet agent. Thus, 261 spontaneous reports involving patients aged ≥65 were included for analysis.

The suspected ADRs reports were grouped in terms of system organ class (SOC) of the Medical Dictionary for Regulatory Activities (MedDRA) [18]. A deeper analysis of the SOCs gastrointestinal, renal and cardiac disorders considering the preferred term reactions (PT) of the MedDRA dictionary was performed; in addition, suspected ADRs that resulted in life-threatening, caused patient hospitalization or prolonged hospitalization were also analyzed in detail. Concerning, these serious outcomes the concomitant drugs were analyzed for gastrointestinal events occurred. In the reports with a fatal outcome, a deeper analysis in terms of each ADR was also performed. The relationship between exposure and death followed the criteria adopted by the PPS, and the World Health Organization-Uppsala Monitoring Center (WHO-UMC) system for case causality assessment [19]. According to this method, which considers the clinical-pharmacological aspects of the reported history and the quality of the documentation reported, the causality is classified as certain, probable, possible, unlikely, conditional or unclassifiable [19].

Considering the seriousness, the reports were grouped as serious or not serious. According to the Guidelines on Pharmacovigilance for Medicinal Products for Human Use, a serious ADR is defined as an adverse reaction that results in death or is life-threatening, causes patient hospitalization or prolonged hospitalization, permanent or significant disability, or birth defect(s) [20].

The descriptive statistical analysis of the data was performed using Microsoft Office Excel 365 Pro Plus.

## 3. Results

### 3.1. Comprehensive Review

From the literature, we identified only 14 articles which were considered eligible for our analysis according to the criteria described in the methods. The years of the selected studies were 2019 (*n* = 1), 2018 (*n* = 2), 2017 (*n* = 1), 2014 (*n* = 1), 2013 (*n* = 1), 2012 (*n* = 3), 2010 (*n* = 1), 2009 (*n* = 1), 2008 (*n* = 1), 2007 (*n* = 1) and 2006 (*n* = 1). Of the 14 studies included in the review, 9 were clinical trials and involved the NSAIDs naproxen, diclofenac, celecoxib and etoricoxib, and the other 5 were observational studies and included several NSAIDs. One of these studies showed the results for elderlies with more than 75 years old. Table 1 shows the type of study, the drugs involved, the number of patients and the outcomes for each study.

#### Main Points Evidenced by the Literature Review

A summary analysis of the studies collected by the search strategy applied in this comprehensive review is presented in Table 1.

Overall, the studies analyzed showed that patients treated with COX-2-selective NSAIDs had a lower risk of adverse gastrointestinal events than those treated with conventional NSAIDs [22], and the concomitant use of gastroprotective agents also lowered the possibility of suffering from the referred adverse effects [23,26]. However, it was also demonstrated that the risk of gastrointestinal events was higher in persons aged ≥75 years taking COX-2-selective NSAIDs when compared with younger patients [23]. On the other hand, a study comparing etoricoxib with diclofenac in patients with osteoarthritis (OA) or rheumatoid arthritis (RA) showed that there was no significant difference between etoricoxib and diclofenac in the development of complicated upper gastrointestinal events [29]. However, a prior lower gastrointestinal tract event and older age significantly increase this risk [29,31]. Advanced age (aged ≥75) and patients with pre-existing renal impairment also rise the incidence of relevant renal adverse events (AEs), such as acute renal failure or decreased urinary output [22,25].

Additionally, the concomitant use of other drugs by older individuals can increase the risk of gastrointestinal bleeding, mainly those controlling hemostasis, such as acetylsalicylic acid, rivaroxaban, clopidogrel and warfarin [3]. However, the use of low-dose acetylsalicylic acid for cardiovascular protection was less likely to be associated with hospitalization when concomitantly taken with celecoxib than with conventional NSAIDs [32].

A study concluded that the rates of thrombotic cardiovascular events in patients with arthritis taking etoricoxib are similar to those in patients on diclofenac after long-term use of these drugs [33]. However, for the same drugs the rates of upper gastrointestinal events were lower with etoricoxib than with diclofenac, but similar for complicated upper gastrointestinal tract events [33].

The incidence of gastrointestinal and cardiovascular AEs was lower with coxibs than with conventional NSAIDs and celecoxib was associated to a lower incidence of these events than etoricoxib. Despite the advanced age and drug exposure time can increase cardiovascular events [30], a study concluded that there was no apparent rise in cardiovascular risk in the celecoxib group when compared with the conventional NSAID group in patients with RA or OA [24].

On the other hand, and as expected, it was evidenced that a topical formulation of diclofenac is well tolerated in persons aged 75 years or older [27,28].

### 3.2. Adverse Drug Reactions Reports Sent to Portuguese Pharmacovigilance System

**Figure 1:**
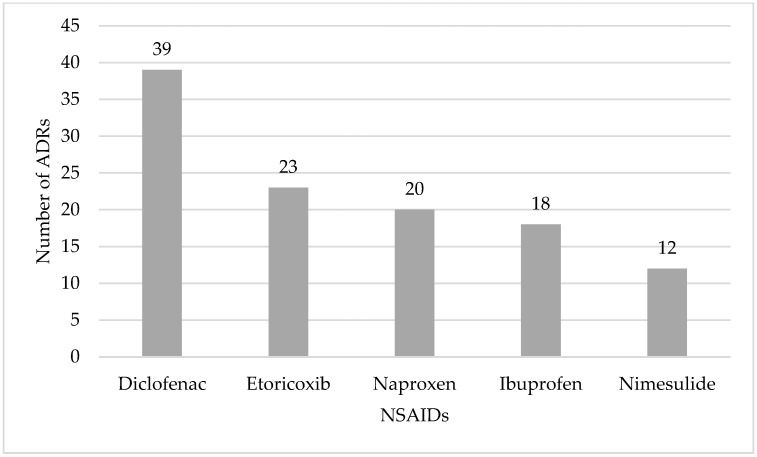
Single non-steroidal anti-inflammatory drugs (NSAIDs) involved in suspected adverse drug reaction (ADR) reports spontaneously reported to the Portuguese Pharmacovigilance System from 2008 to 2018 for people aged 65 years or older: the top five.

In our study we found 261 reports associated to NSAIDs, for people aged 65 years or older. The number of reports associated to NSAIDs, are presented in Figure 1.

The mostly reported NSAIDs as a single suspected drug associated to ADRs were diclofenac (39 reports) followed by etoricoxib (27 reports).

**Figure 2:**
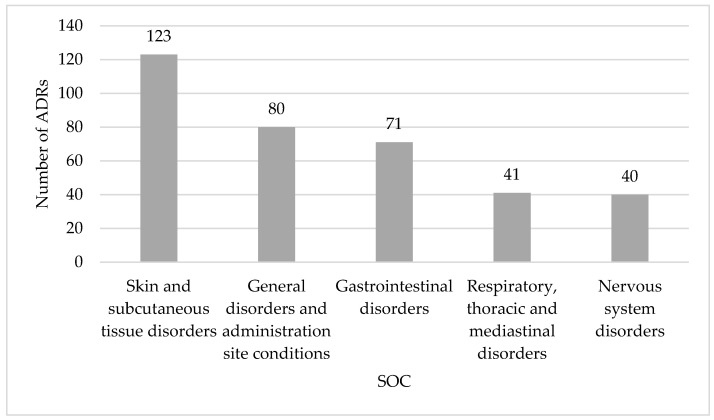
System organ classes (SOC) affected by suspected adverse drug reactions (ADR) reports spontaneously reported to the Portuguese Pharmacovigilance System from 2008 to 2018 for people aged 65 years or older: the top five.

In 180 reports (69.0%) only one NSAID was referred as the suspected drug, but in 71 reports (27.2%) a NSAID was associated to other drug classes, and in 10 reports (3.8%) associations between NSAIDs were detected. Most suspected ADRs occurred in females (64.7%, *n* = 169) and a high percentage the suspected ADRs were serious (71.3%, *n* = 186). Nearly 59.8% (*n* = 156) of the reports analyzed belong to the age group of 65 to 74 years, followed by 31.8% (*n* = 83) of the age group 75 to 84 years and 8.4% (*n* = 22) concerned patients aged 85 and older. The SOC “skin and subcutaneous tissue disorders” was the mostly reported, followed by “general disorders and administration site conditions” and “gastrointestinal disorders” (Figure 2).

A deeper analysis of the SOC “gastrointestinal disorders” showed that 66.2% (*n* = 47) reports were serious, 1 resulted in death, 6 were life-threatening, and 11 caused patient hospitalization or prolonged hospitalization. Among the reports associated to life-threatening adverse events that caused patient hospitalization or prolonged hospitalization, gastrointestinal hemorrhage mainly occurred in patients who had taken two or more NSAIDs and/or anticoagulant agents. Ibuprofen and diclofenac were the drugs most commonly associated with gastrointestinal events (Table 2). The gastroprotection only was presented in five reports, but in five reports the concomitant drugs were unknown.

From a deeper analysis of the SOC “renal and urinary disorders” identified in 19 reports, it was found that 17 were serious, 1 resulted in death, 2 in life-threatening adverse events and 7 caused patient hospitalization or prolonged hospitalization. In these reports, only 2 patients were aged <74 years. Ibuprofen and naproxen were the drugs most associated with renal injury or renal failure, but 3 of these patients were diabetic. The patient that suffered aggravated chronic kidney disease associated to diclofenac had clinical history of chronic kidney disease (Table 3). The SOC “cardiac disorders” only had 7 reports associated, but 6 were serious, 2 resulted in death, 1 was life-threatening and 1 led to hospitalization (Table 3). A deeper analysis of the reports of these 2 patients (1 was life-threatening and 1 was hospitalized) allowed to conclude that they had clinical history of arterial hypertension.

A deeper analysis of the serious reports with fatal outcome was also performed. The patients died in 7 cases and the drugs involved were naproxen and dabigatran etexilate, strontium ranelate and etoricoxib, acetylsalicylic acid, diclofenac and ibuprofen, diclofenac and thiocolchicoside and diclofenac combinations and allopurinol (Table 4).

Only in two of these reports was a single NSAID involved as the suspected drug. In the remainder reports the NSAID was associated with another drug. However, after evaluation of the reports according to the WHO system for standardized causality assessment of cases as described in the Methods Section, melaena and gastrointestinal hemorrhage were considered as probably related to the use of dabigatran etexilate. In the other reports, only the combination of strontium ranelate and etoricoxib and the combinations of diclofenac and thiocolchicoside were considered as possible causative agents of the respective adverse reaction. For the remaining reports, the causality assessment was not presented.

## 4. Discussion

Considering the analysis of data obtained from the literature review and from the suspected ADRs reported to PPS in order to assess the safety use of NSAIDs in older patients we concluded that, in general, coxibs showed to be safer than conventional NSAIDs [3,23,26,30,34]. However, clinical monitoring of the risks and potential adverse events (mainly gastrointestinal effects) should be mitigated with reduction of concomitant drugs use, if possible, and use of gastroprotection. Regarding the reports sent to the PPS, in 69.0% of them only a single NSAID was the suspected drug, and the majority of reports were considered serious, associated to the female gender and belonging to the age group of 65 to 74 years, similar to the observed in other analogous studies [8,35,36]. Even though etoricoxib was one of the five most reported drugs to the PPS, the more classical diclofenac, naproxen, ibuprofen, and nimesulide remained the mostly reported drugs. Despite acetylsalicylic acid was not in the five most reported, we found two fatal outcomes associated to this drug, and in one of them the patient suffered a cerebrovascular accident. In this context, a meta-analysis demonstrated a trend towards increased risk of hemorrhagic stroke and a 50% relative risk increase of major gastrointestinal bleeding in acetylsalicylic acid users [37]. In addition, the gastrointestinal bleeding risk increased when taking acetylsalicylic acid and rivaroxaban with other NSAIDs. Therefore, it is important to reduce NSAIDs use by older adults, especially acetylsalicylic acid, and avoid rivaroxaban in older persons taking NSAIDs [3].

Additionally, the concomitant use of NSAIDs and proton pump inhibitor (PPIs) reduced the gastrointestinal perforation, ulcers, or bleeding (PUB) [23]. In fact, when compared with the use of isolated conventional NSAIDs, the risk of PUB was lower for those aged ≥75 years taking conventional NSAIDs with PPIs [23]. Another study concluded that the incidence of gastrointestinal events was lower for coxibs than for conventional NSAIDs and that celecoxib was associated to a lower incidence than etoricoxib [30]. However, patients with advanced age and higher drug exposure time had a significantly increased risk of gastrointestinal events, which can be reduced with the use of gastroprotective agents [30]. In general, the discontinuation of the treatment due to AE is higher with conventional NSAIDs [26]. Prior dyspepsia or upper gastrointestinal events and age ≥ 65 years were associated to an increased risk of developing dyspepsia, severe enough to led to NSAIDs discontinuation [29]. In fact, a study showed that the risk of an upper gastrointestinal clinical event with NSAID use is not statistically significant when comparing the COX-2-selective inhibitor etoricoxib with the traditional NSAID diclofenac, but this risk increases with a prior gastrointestinal event [31]. Our analysis of the reports showed that the drug with most reports was a conventional NSAID and the SOC “gastrointestinal disorders” was one of the most reported. Additionally, the gastrointestinal hemorrhage (which includes the cases of melaena reported) occurred in patients with concomitant treatments and not all patients had appropriate gastroprotection. Despite it was already clearly demonstrated that antiplatelets, anticoagulants and the concomitant use of NSAIDs increase the risk of gastrointestinal bleeding [1,11,14], we found in our study that several patients were taking combinations of these drug families. In this context, ibuprofen, which is considered safe in this population due to its short half-life, in some reports was associated with other drugs that increase gastrointestinal adverse events [11]. Indeed, it is recommended to prescribe gastroprotective agents to older patients taking NSAIDs [13]. In fact, the STOPP/START criteria, used for medication review in the elderly, considered that NSAID with concurrent antiplatelet agent(s) without PPI prophylaxis increased the risk of peptic ulcer disease [38], so it is essential that these patients take gastroprotective agents in certain specific circumstances, and in real-world, this is not always the case.

Among elderly arthritis patients, the incidence of gastrointestinal intolerability was lower with celecoxib than with naproxen, ibuprofen, or diclofenac. In general, the elderly patients that discontinued NSAIDs use due to gastrointestinal intolerability were using naproxen or ibuprofen [39]. In fact, analyzing the reports to the PPS, the drugs most reported were diclofenac, naproxen, ibuprofen and nimesulide. In this context there were, also, reported to PPS associated to these drugs diarrhea, nausea, vomiting and abdominal pain, as non-serious ADRs, and serious ADRs, as gastrointestinal hemorrhages.

Gastrointestinal events, including bleeding and ulceration, increase in frequency and seriousness with increasing age [40]. Old patients receiving NSAIDs are also more susceptible to renal side effects, including renal vasoconstriction and increased tubular sodium reabsorption that may cause fluid retention, oedema and worsening of congestive cardiac failure [40]. In this context, most NSAIDs can also contribute to worsening of chronic renal failure, particularly in patients with co-existing renal damage or patients taking diuretics or angiotensin converting enzyme inhibitors [40]. Concerning our data, the patients that suffered renal disorders were diabetics, a major risk factor for kidney disease [41] or had clinical history of chronic renal failure and the majority also had age ≥ 74. The results were in agreement with the literature that refer that advanced age (aged ≥ 75) and patients with pre-existing renal impairment rise the incidence of relevant renal AEs [22,25]. Despite this, in our study, the estimated glomerular filtration rate (eGFR) is not available. However, the STOPP/START criteria consider that the use of NSAID’s if eGFR < 50 mL/min/1.73 m^2^ increase the risk of deterioration in renal function [38].

In our study using data from PPS, the reports with fatal outcome (Table 3) associated to diclofenac users included blood pressure immeasurable and hypotension. Despite these ADRs were associated to the diclofenac and thiocolchicoside, the AEs are in agreement with three phase III clinical trials that studied one or more doses of HPbCD-diclofenac or placebo in older patients with acute moderate-to-severe postoperative pain [22]. These authors concluded that the incidences of postoperative anemia, constipation, and hypotension increased significantly across the age groups [22]. In fact, it was evidenced that NSAIDs administration may produce an increase in a mean arterial blood pressure of 5 mmHg [42]. Also, with respect to diclofenac, the relative risks were similar in the diclofenac and placebo groups for all studied SOC categories and preferred terms [22]. The SOC category of ‘Gastrointestinal disorders’ were the most common, and this was driven predominantly by cases of nausea and constipation [22]. In our study we also found acute kidney injury in one report associated to this drug. In this context, Chelly et al. found a significantly higher incidence of acute renal failure in those aged ≥75 years [22]. Other study showed that intravenous (IV) HPbCD diclofenac is safe and well tolerated. However, elevated incidences of relevant renal AEs (acute renal failure, decreased urinary output) were again observed in patients >75 years as well as in those with significant pre-existing renal impairment [25]. A meta-analysis performed by Ungprasert et al. demonstrated that exist an elevated acute kidney injury risk in patients taking conventional NSAIDs [43].

A study performed by Couto et al. concluded that naproxen can be relatively safe in younger and older patients [21]. The ADRs most described for this drug were related to the gastrointestinal system, but the study also showed that there was no significant differences in AE between groups, regardless of age [21]. In our analysis of ADR reported to PPS, in the fatal outcome associated to this drug, the patient suffered melena and hemorrhage, which is expected due to its mechanism of action [5]. However, the patient was also taking dabigatran etexilate, whereby the authority considered that the ADRs reported were related to this drug. Despite dabigatran is considered an anticoagulant with a good safety profile, its use also requires considerable caution, particularly in elderly, high bleeding risk patients, patients with decreased renal function and those on complex drug regimens [37]. Among patients receiving antithrombotic therapy after myocardial infarction, the use of NSAIDs was associated with an increased risk of bleeding and thrombotic events, even after short-term treatment [44]. Another study concluded that in elderly patients receiving cardiovascular protection with low- acetylsalicylic acid and pain control with NSAIDs, celecoxib may be safer with regards to gastrointestinal toxicity than conventional NSAIDs [32].

Concerning the cardiovascular risk for serious AE (such as myocardial infarction, angina pectoris heart failure) a study showed that there was no apparent increase in cardiovascular risk in the celecoxib group when compared with the NSAID group in patients with RA or OA with a higher risk of cardiovascular disease [24]. In addition, a retrospective cohort study concluded that the incidence of serious cardiovascular events was lower for coxibs than for NSAIDs and celecoxib was associated to a lower incidence than etoricoxib [30]. However, the female gender, advanced age, and drug exposure time significantly affected cardiovascular events [30]. Another study for etoricoxib vs diclofenac for cardiac events, cerebrovascular events, and peripheral vascular events did not show any discernible difference between treatment groups [33]. The same study concluded that the rates of thrombotic cardiovascular events in patients with arthritis on etoricoxib are similar to those in patients on diclofenac with long-term use of these drugs [33]. The most serious cardiovascular disorders, in our study, were found in patients who were taking other drugs and had clinical history of hypertension, so most predisposed for these events.

Although topical NSAIDs are considered safer and well-tolerated in older patients than oral NSAIDs (fewer severe gastrointestinal events), a substantial proportion of older adults reports systemic AEs with topical agents [15]. The most common AE for topical diclofenac involved the skin or subcutaneous tissue [27]. Actually, the SOC most reported to the PPS was skin disorders. In fact, NSAIDs use are one of the leading causes of hypersensitivity reactions to drugs by nonspecific or by specific immunological mechanisms [45]. In fact, these drugs may be responsible for exacerbated respiratory disease, cutaneous disease, urticaria/angioedema or anaphylaxis [46], which can explain the ADRs associated to the fatal outcome for diclofenac and for acetylsalicylic acid (Table 3) and the fact that the SOC “skin and subcutaneous” was the mostly reported.

Concerning the fatal outcome associated to strontium ranelate and etoricoxib, where the ADRs reported were fatigue, pulmonary hypertension, pneumonia, respiratory failure we only suspect that they maybe be related with other comorbidities. In this context an increased risk for cardiac events with strontium ranelate was also described [47]. However, the fatigue can be explained by other diseases. It is also important to mention that elderlies present a progressive decrease in immune function and, consequently, increased susceptibility to infectious disease, namely pneumonia [48].

Despite some limitations, this study performed an assessment of the safety of NSAIDs in the elderly. The main weakness refers to the diversity of different studies selected for the review, which difficult the comparisons between them. Data were obtained from clinical trials or observational studies where patients had different inclusion and exclusion criteria. Different doses of NSAID were tested, and not all studies had a placebo group. The outcomes measured in each trial were also different, which may bias the results. Additionally, data related to the PPS must be interpreted cautiously, as a fatal outcome does not necessarily imply a causal relationship with the suspected drug. The lack of information in most reports, makes it is impossible to perform a correct causality assessment and attribute an ADR to a drug. It is also important to mention that several health professionals believe that if the ADR is known for some drug it is not necessary to report it [49,50]. This point can explain the few reports associated to NSAIDs in the PPS and also the relatively low number of reports with gastrointestinal events.

## 5. Conclusions

The use of NSAIDs as a drug therapy for a wide range of conditions is increasing, in part due to the increase of elderly population, raising the risk of AE. Therefore, a selection of an appropriate NSAID taking in consideration the risk-benefit factors is very important in the elderly. The advanced age and the use of other concomitant drugs were associated to an increased risk of adverse events. In addition, the use gastroprotective agents, that can decrease some of these risks, is not always observed in real-world. For all NSAIDs it is important to evaluation of the renal function since with age ≥ 75 an increase of acute renal failure occurrences was observed, increasing the AE risk.

The results showed that even though NSAIDs’ toxicity is well understood, their safe use need to be monitored in clinical practice, so it is urgent to increase the appropriateness of the medication regimen to improve the quality of pharmacotherapy of this special population.

## Figures and Tables

**Table 1:** Studies evaluating the safety of non-steroidal anti-inflammatory drugs (NSAIDs) in the elderly.

Reference	Study Design	Study Population	Number of Patients with ≥65 Years Old	Number of Patients with <65 Years Old	Drugs Compared/Route of Administration	Outcomes
Dillon et al., 2019 [3]	Retrospective observational study for AE reported to Food and Drug Administration’s Adverse Events Reporting System	Patients with an NSAID as the primary suspect for an AE	*n* = 1347	*n* = 0	Acetylsalicylic acid, naproxen, ibuprofen, diclofenac, celecoxib or other NSAID; oral	• 72.5% of the AEs were associated to acetylsalicylic acid. • Predictors of gastrointestinal bleed: ➢ Acetylsalicylic acid ➢ Rivaroxaban ➢ Concurrent NSAID
Couto et al., 2018 [21]	Four multicenter, multidose, randomized, parallel, double-blind, placebo-controlled studies	Patients with OA	*n* = 229, placebo *n* = 231	*n* = 180, placebo *n* = 178	Naproxen/placebo; oral	Rate of AEs and gastrointestinal events comparable in the naproxen sodium and placebo groups (26% and 24% of patients, and 13% vs. 10%, respectively) Most common AEs were related to the gastrointestinal system and similar in two groups (dyspepsia, nausea, and diarrhea)
Chelly et al., 2018 [22]	Three phase III trials (2 were randomized, placebo- and active controlled trials and 1 was open-label, multiple-dose safety study)	Patients with Acute Moderate-to-Severe Postoperative Pain	*n*= 411	*n* = 878	One or more doses of HPbCD-diclofenac or placebo; injectable	Incidence of AE similar in the groups Gastrointestinal disorders were the most common AE Higher incidence of acute renal failure in those aged ≥75 years (3.9%) than was observed in those aged <65 years (0.1%) or 65–74 years (0.4%)
Bakhriansyah et al., 2017 [23]	Case–control study with data obtained from the Dutch PHARMO Record Linkage System	Patients with a first hospital admission for risk of gastrointestinal perforation, ulcers, or bleeding (PUB)	Age ≥ 75 *n* = 2890, control 2184	18–74 *n* = 1504, control 2890	Conventional NSAIDs or selective COX-2, alone or combined with PPI; route of de administration unknown	Selective COX-2 inhibitors combined with PPIs had the lowest risk of PUB followed by selective COX-2 inhibitors and conventional NSAIDs with PPIs Risk of PUB was lower for those aged ≥75 years taking conventional NSAIDs with PPIs compared with younger patients with conventional NSAIDs. Risk of PUB, for those aged ≥75 years taking selective COX-2 inhibitors, was higher compared with younger patients
Hirayama et al., 2014 [24]	Prospective, nonblinded, non-randomized, comparative observational study performed in hospitals and general practice clinics	Patients with OA or RA	Celecoxib (*n*= 5591), other NSAIDs (*n* = 5057)	Celecoxib (*n* = 1767), other NSAIDs (*n* = 1692)	Celecoxib/ Others NSAIDS (Loxoprofen, Etodolac, Meloxicam, Lornoxicam, Diclofenac, Zaltoprofen, Other); route of administration unknown	No apparent increase in cardiovascular risk in the celecoxib group compared with the conventional NSAID group.
Chelly et al., 2013 [25]	Multicenter, open-label, repeated dose, multiple-day, single-arm safety study	Patients with acute moderate-to-severe pain following major surgery	*n* = 367	*n* = 604	HPbCD diclofenac; injectable	Elevated incidences of renal AEs (acute renal failure, decreased urinary output) in patients >75 years of age and in those with significant pre-existing renal impairment.
Kellner et al., 2012 [26]	Prospective, double blind, randomized, parallel-group, multicenter, international study	Patients with OA and/or RA	*n* = 2446	*n* = 0	Celecoxib or diclofenac slow release 75 mg plus omeprazole 20 mg once a day; route of administration unknown	Incidence of gastrointestinal events in the celecoxib group was lower compared with the diclofenac group Incidence of moderate-to-severe abdominal symptoms and discontinuation of treatment due to gastrointestinal AEs were lower in the celecoxib group. Celecoxib was shown to be superior to a conventional NSAID plus a PPI in reducing the risk of clinical outcomes across the entire gastrointestinal tract.
Roth et al., 2012 [27]	Seven multicenter, randomized, blinded, PhaseIII clinical trials	Patients ≥75 years with a primary diagnosis of OA in the knee or hand	*n* = 280	*n* = 0	TDiclo solution 1.5% [*w*/*w*] in 45.5% dimethyl sulfoxide; placebo (topical lotion consisting of 2.33% or 4.55% dimethyl sulfoxide); and control (topical lotion consisting of 45.5% dimethyl sulfoxide); topical	Skin or subcutaneous tissue were the most AE reported Few patients (18%) reported gastrointestinal AE (constipation, diarrhea, and nausea were the most common AE). Cardiovascular and renal/urinary AE were rare, and group differences were not detected.
Baraf et al., 2012 [28]	Five randomized, double-blind, placebo-controlled trials	Patients with mild to moderate OA of the knee and hand	*n* = 538	*n* = 888	Diclofenac sodium gel (DSG) or placebo (vehicle gel); topical	Similar and low rates of AEs in DSG-treated patients aged ≥65 and <65 years
Laine et al., 2010 [29]	Three double-blind randomized trials	Patients OA or RA	*n* = 14,227	*n* = 20,474	Etoricoxib or diclofenac; oral	• Predictors of clinical events and complicated events: ➢ age ≥ 65 ➢ prior event ➢ low-dose acetylsalicylic acid ➢ corticosteroid • Predictors of discontinuation due to dyspepsia: ➢ prior dyspepsia ➢ prior event ➢ age ≥ 65 years • No significant difference for etoricoxib vs. diclofenac in the complicated upper gastrointestinal events
Turajane et al., 2009 [30]	Hospital-based retrospective cohort study	Patients with knee OA	*n* = 1030	*n* = 0	Conventional NSAIDs (diclofenac, diflunisal, sulindac, piroxicam, indomethacin, loxoprofen, meloxicam, nimesulide, and naproxen) or two coxibs (celecoxib and etoricoxib); oral	• Incidence of gastrointestinal and cardiovascular events was lower for coxibs than for conventional NSAIDs • Patients with advanced age and higher drug exposure time had a significantly increased risk of gastrointestinal events • The use of gastroprotective agents significantly decreased gastrointestinal risks • Factors that significantly increase the risk of cardiovascular events: ➢ females ➢ age > 80 years ➢ drug exposure time
Laine et al., 2008 [31]	Three randomized, double-blind, clinical trials	Patients with OA or RA	*n* = 14,396	*n* = 20,305	Etoricoxib or diclofenac; oral	• There is not a statistically significant decrease in lower gastrointestinal clinical events with the COX-2 selective inhibitor etoricoxib versus the traditional NSAID diclofenac • The major risk factors of lower gastrointestinal events are: ➢ a prior gastrointestinal event ➢ age > 65 years
Rahme et al., 2007 [32]	Retrospective cohort study using Quebec government databases	Patients ≥ 65 who filled a prescription for celecoxib or a conventional NSAID	*n* = 332,491	*n* = 0	Conventional NSAID only, celecoxib only, conventional NSAID and low-dose acetylsalicylic acid, celecoxib and acetylsalicylic acid; oral	Celecoxib without acetylsalicylic acid was less likely than conventional NSAID without acetylsalicylic acid to be associated with gastrointestinal hospitalization Celecoxib and acetylsalicylic acid were also less likely to be associated with gastrointestinal hospitalization than conventional NSAID and acetylsalicylic acid Gastrointestinal hospitalization rates were similar for celecoxib and acetylsalicylic acid and conventional NSAID without acetylsalicylic acid
Cannon et al., 2006 [33]	Three randomized, double-blind clinical trials	Patients with OA or RA	*n* = 14,396	*n* = 20,305	Etoricoxib or diclofenac; oral	Rates of thrombotic cardiovascular events are similar for etoricoxib and for diclofenac Rates of upper gastrointestinal clinical events (perforation, bleeding, obstruction, ulcer) were lower with etoricoxib than with diclofenac Rates of complicated upper gastrointestinal events were similar for etoricoxib and diclofenac

AE, adverse event; COX-2, cyclooxygenase-2; DSG, diclofenac sodium gel; HPbCD-diclofenac, hydroxypropyl-b-cyclodextrin-diclofenac; NSAID, non-steroidal anti-inflammatory drug; NSAIDs, non-steroidal anti-inflammatory drugs; OA, osteoarthritis; PPI, proton pump inhibitor; PUB, gastrointestinal perforation, ulcers, or bleeding; RA, rheumatoid arthritis; TDiclo, diclofenac sodium topical solution 1.5% (*w*/*w*) in 45.5% dimethyl sulfoxide.

**Table 2:** Non-steroidal anti-inflammatory drugs and other drug classes associated with serious gastrointestinal events that resulted in life-threatening adverse events, caused patient hospitalization or prolonged hospitalization.

Suspected Drugs	Number of Occurrences	Preferred Terms	Concomitant Drugs
Aceclofenac	1	Melaena, Duodenal ulcer, Gastric ulcer	Unknown
Acetylsalicylic acid + diclofenac ^b^	1	Gastrointestinal hemorrhage	Unknown
Dabigatran etexilate + etoricoxib + amiodarone ^b^	1	Melaena, Hematochezia	Furosemide + glyceryl trinitrate + tramadol and paracetamol ^a,b^
Dexketoprofen	1	Vomiting	Paracetamol + tramadol + etoricoxib + pregabalin ^b^
Diclofenac	2	Tongue oedema, Diarrhoea, Melaena, Gastric haemorrhage, Erosive oesophagitis	Unknown
Diclofenac + colchicine ^b^	1	Diarrhea, Nausea, Dyspepsia, Vomiting, Abdominal pain upper	Salbutamol
Escitalopram + ibuprofen ^b^	1	Gastric ulcer, Rectal injury, Abdominal pain, Decreased appetite, Rectal hemorrhage, Hematemesis	Metamizole + acetylsalicylic acid + sucralfate + pravastatin and fenofibrate ^a^ + citicoline + furosemide + etoricoxib + ferrous sulfate + midazolam + spironolactone ^b^
Etoricoxib	1	Abdominal distension	Amiodarone + pantoprazole + diazepam + levomepromazine ^b^
Ibuprofen + nimesulide ^b^	1	Hematochezia	Omeprazole + alprazolam + pravastatin + amitriptyline + acetylsalicylic acid 100 mg + gabapentin + potassium clorazepate + metamizole ^b^
Ibuprofen	1	Lip oedema	Unknown
Imidapril + ketoprofen ^b^	1	Tongue oedema	Cobamamide + lansoprazole + atorvastatin + furosemide + finasteride + gliclazide + acetylsalicylic acid 100 mg + allopurinol + rilmenidine + idebenone ^b^
Indomethacin	1	Gastrointestinal hemorrhage	Ibuprofen +clopidogrel ^b^
Metformin and vildagliptin ^a^ + acemetacin ^b^	2	Vomiting, Diarrhea	Simvastatin + furosemide +perindopril + amlodipine and indapamide ^a^ + sertraline + trazodone + codeine + lorazepam + omeprazole + magnesium + allopurinol ^b^
Proglumetacin + metamizole ^b^	1	Diarrhea, Nausea	Pregabalin
Ribavirin + sofosbuvir and ledipasvir ^a^ + ibuprofen ^b^	1	Duodenal ulcer hemorrhage	Unknown

^a^ Fixed combination of drugs (patient taking only a medication). ^b^ Combinations of two or more different medications (patient taking different medication each one containing a chemical substance).

**Table 3:** Non-steroidal anti-inflammatory drugs and other class of drugs associated to serious renal events and cardiac disorders where occurred adverse reaction that resulted in life-threatening adverse event, caused patient hospitalization or prolonged hospitalization.

SOC	Preferred Term	Suspected Drugs
Renal and urinary disorders	Acute renal failure	Etoricoxib + ciprofloxacin ^b^
	Tubulointerstitial nephritis	Ibuprofen
	Renal injury	Metformin + ibuprofen ^b^
	Renal failure chronic aggravated	Diclofenac + colchicine ^b^
	Haematuria	Warfarin + diclofenac ^b^
	Renal failure acute	Ibuprofen
	Chronic kidney disease	Naproxen
	Acute renal insufficiency	Naproxen + metformin and vildagliptin ^a,b^
	Tubulointerstitial nephritis	Carvedilol + ibuprofen + allopurinol + carvedilol ^b^
Cardiac disorders	Tachycardia, blood pressure increased	Etofenamate + diclofenac + thiocolchicoside ^b^
	Cardio-respiratory arrest	Acetylsalicylic acid

^a^ Fixed combination of drugs (patient taking only a medication). ^b^ Combinations of two or more different medications (patient taking different medication each one containing a chemical substance).

**Table 4:** Adverse Drug Reactions (ADR) and suspected drugs associated with a fatal outcome.

Drugs	ADR Preferred Term (PT)
Naproxen + dabigatran etexilate ^b^	Melaena, Gastrointestinal haemorrhage
Strontium ranelate + etoricoxib ^b^	Fatigue, Pulmonary hypertension, Pneumonia, Respiratory failure
Acetylsalicylic acid	Cerebrovascular accident
Acetylsalicylic acid	Hypotension, Acute respiratory failure, Tracheobronchitis, Cardiac failure, Prinzmetal angina
Diclofenac + ibuprofen ^b^	Acute kidney injury
Diclofenac + thiocolchicoside ^b^	Blood pressure immeasurable, Bronchospasm, Respiratory distress, Hypotension, Hypoxia, Oxygen saturation decreased, Sinus tachycardia, Rash, Hyperhidrosis
Diclofenac combinations + allopurinol ^b^	Thermal burn

^b^ Combinations of two or more different medications (patient taking different medication each one containing a chemical substance).

## Data Availability

The raw data used in this research are available to the authors, depending on INFARMED’s authorization.
